# Aurora B but Not Rho/MLCK Signaling Is Required for Localization of Diphosphorylated Myosin II Regulatory Light Chain to the Midzone in Cytokinesis

**DOI:** 10.1371/journal.pone.0070965

**Published:** 2013-08-07

**Authors:** Tomo Kondo, Rieko Isoda, Takayuki Ookusa, Keiju Kamijo, Kozue Hamao, Hiroshi Hosoya

**Affiliations:** 1 Department of Biological Science, Graduate School of Science, Hiroshima University, Higashi-Hiroshima, Hiroshima, Japan; 2 Department of Anatomy and Anthropology, Graduate School of Medicine, Tohoku University, Sendai, Miyagi, Japan; Astar-Neuroscience Research Partnership (NRP) and Institute of Medical Biology (IMB), Singapore

## Abstract

Non-muscle myosin II is stimulated by monophosphorylation of its regulatory light chain (MRLC) at Ser19 (1P-MRLC). MRLC diphosphorylation at Thr18/Ser19 (2P-MRLC) further enhances the ATPase activity of myosin II. Phosphorylated MRLCs localize to the contractile ring and regulate cytokinesis as subunits of activated myosin II. Recently, we reported that 2P-MRLC, but not 1P-MRLC, localizes to the midzone independently of myosin II heavy chain during cytokinesis in cultured mammalian cells. However, the mechanism underlying the distinct localization of 1P- and 2P-MRLC during cytokinesis is unknown. Here, we showed that depletion of the Rho signaling proteins MKLP1, MgcRacGAP, or ECT2 inhibited the localization of 1P-MRLC to the contractile ring but not the localization of 2P-MRLC to the midzone. In contrast, depleting or inhibiting a midzone-localizing kinase, Aurora B, perturbed the localization of 2P-MRLC to the midzone but not the localization of 1P-MRLC to the contractile ring. We did not observe any change in the localization of phosphorylated MRLC in myosin light-chain kinase (MLCK)-inhibited cells. Furrow regression was observed in Aurora B- and 2P-MRLC-inhibited cells but not in 1P-MRLC-perturbed dividing cells. Furthermore, Aurora B bound to 2P-MRLC *in vitro* and *in vivo*. These results suggest that Aurora B, but not Rho/MLCK signaling, is essential for the localization of 2P-MRLC to the midzone in dividing HeLa cells.

## Introduction

During cytokinesis in animal cells, specific machinery called the contractile ring forms to divide a mitotic cell. Schroeder originally reported that the contractile ring in jellyfish eggs is composed of numerous fine filaments [1]. Treatment with heavy meromyosin identified these filaments as F-actin [2]. Further, it has been reported that myosin II is localized to the contractile ring [3] and required for cytokinesis [4] in cultured mammalian cells and starfish eggs. A consensus opinion has held that the concerted sliding interaction of F-actin and myosin II filaments observed in muscle contraction causes the constriction of the contractile ring during cytokinesis [5], [6]. However, strikingly, filamentous myosin II has not yet been found at the contractile ring. Thus, it is still controversial whether the proposed but unproven sliding model provides a plausible explanation for contractile ring constriction during cytokinesis.

Non-muscle myosin II is a hexamer comprising 2 myosin heavy chains (MHCs), 2 essential light chains, and 2 regulatory light chains (MRLCs) [7], [8]. Phosphorylation of MRLC at Ser19 (1P-MRLC) enhances myosin II activity, which is further enhanced by MRLC diphosphorylation at Thr18/Ser19 (2P-MRLC) *in vitro* [9], [10]. Indirect immunofluorescence observations with a conventional light microscope showed that 1P- and 2P-MRLC localize to the contractile ring in dividing cells [11]–[13]. Using confocal microscopy, we have previously shown that 2P-MRLC, but not 1P-MRLC, also localizes to the midzone in cultured mammalian cells [14]. Interestingly, MHC was not observed at the midzone, suggesting that 2P-MRLC plays a unique role different from its role as a subunit of myosin II. However, the mechanism(s) underlying the localization of 2P-MRLC to the midzone during cytokinesis has not been elucidated.

Here, we demonstrate that a mitotic kinase, Aurora B [15], is required for 2P-MRLC localization to the midzone in dividing HeLa cells. Depleting midzone-localizing Aurora B, but not centralspindlin, a heterotetramer composed of mitotic kinesin-like protein 1 (MKLP1, kinesin-6) and MgcRacGAP (GTPase-activating protein) [16], or the Rho guanine nucleotide exchange factor ECT2, by RNA interference (RNAi) disrupted the midzone localization of 2P-MRLC. An inhibitor of Aurora B also reduced the localization of 2P-MRLC at the midzone. Unexpectedly, 1P-MRLC still localized to the contractile ring in Aurora B-depleted or Aurora B-inhibited cells. Moreover, regression of the cleavage furrow was observed in Aurora B-inhibited cells, suggesting that the Aurora B activity that regulates the localization of 2P-MRLC at the midzone is required for the completion of abscission, a process of severing the intercellular bridge connecting the 2 daughter cells. Further, biochemical analyses showed that Aurora B directly bound 2P-MRLC. Treatment with a myosin light-chain kinase (MLCK) inhibitor had no effect on the localization of 1P-MRLC and 2P-MRLC. Therefore, we conclude that Aurora B, but not Rho/MLCK signaling, plays an important role in 2P-MRLC localization to the midzone in dividing HeLa cells.

## Results

### Specificity of Anti-2P-MRLC Monoclonal Antibody in Immunofluorescence

**Figure 1:**
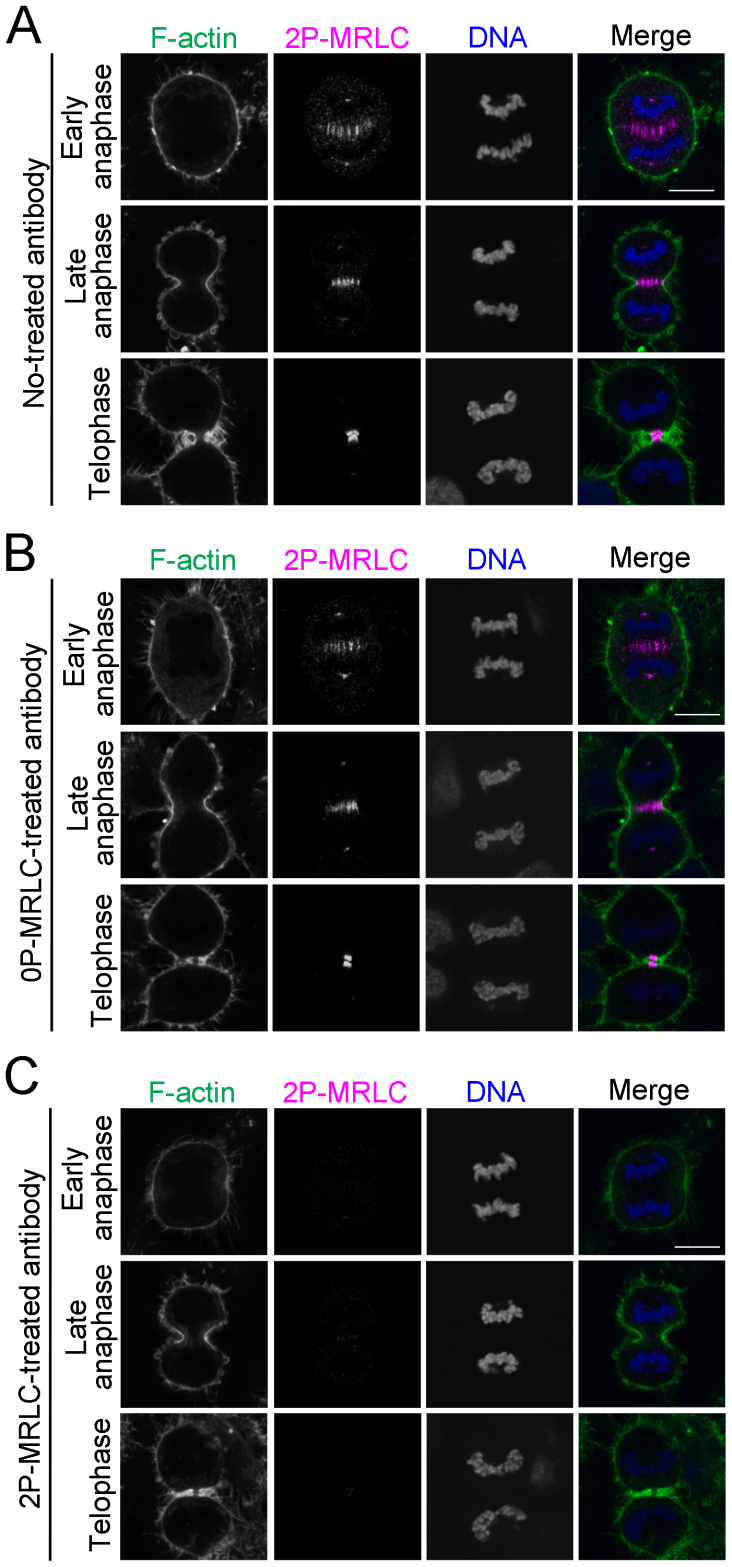
2P-MRLC localizes to the centrosomal region, contractile ring, midzone, and midbody during cytokinesis. HeLa cells were fixed and immunostained with anti-2P-MRLC monoclonal antibody (4F12) preincubated with PBS (A), 0P-MRLC (B), or 2P-MRLC (C) at 37°C for 60 min. Bar, 10 µm.

We have previously reported that an anti-2P-MRLC antibody, 4F12, stains the centrosomal region, contractile ring, midzone, and midbody during cytokinesis in various mammalian cultured cells [14]. In this study, we further investigated the specificity of this antibody at the immunofluorescence level by attempting to absorb its antigen (2P-MRLC). Antibody preincubated with or without 0P-MRLC still immunostained the centrosomal region, midzone, contractile ring, and midbody in dividing HeLa cells (Fig. 1A and B). In contrast, this staining pattern was not observed when we used antibody absorbed by 2P-MRLC (Fig. 1C). These data clearly suggest that the 4F12 antibody has a high specificity for 2P-MRLC in immunofluorescence.

### Rho/MLCK Signaling is not Required for 2P-MRLC Localization to the Midzone during Cytokinesis

**Figure 2:**
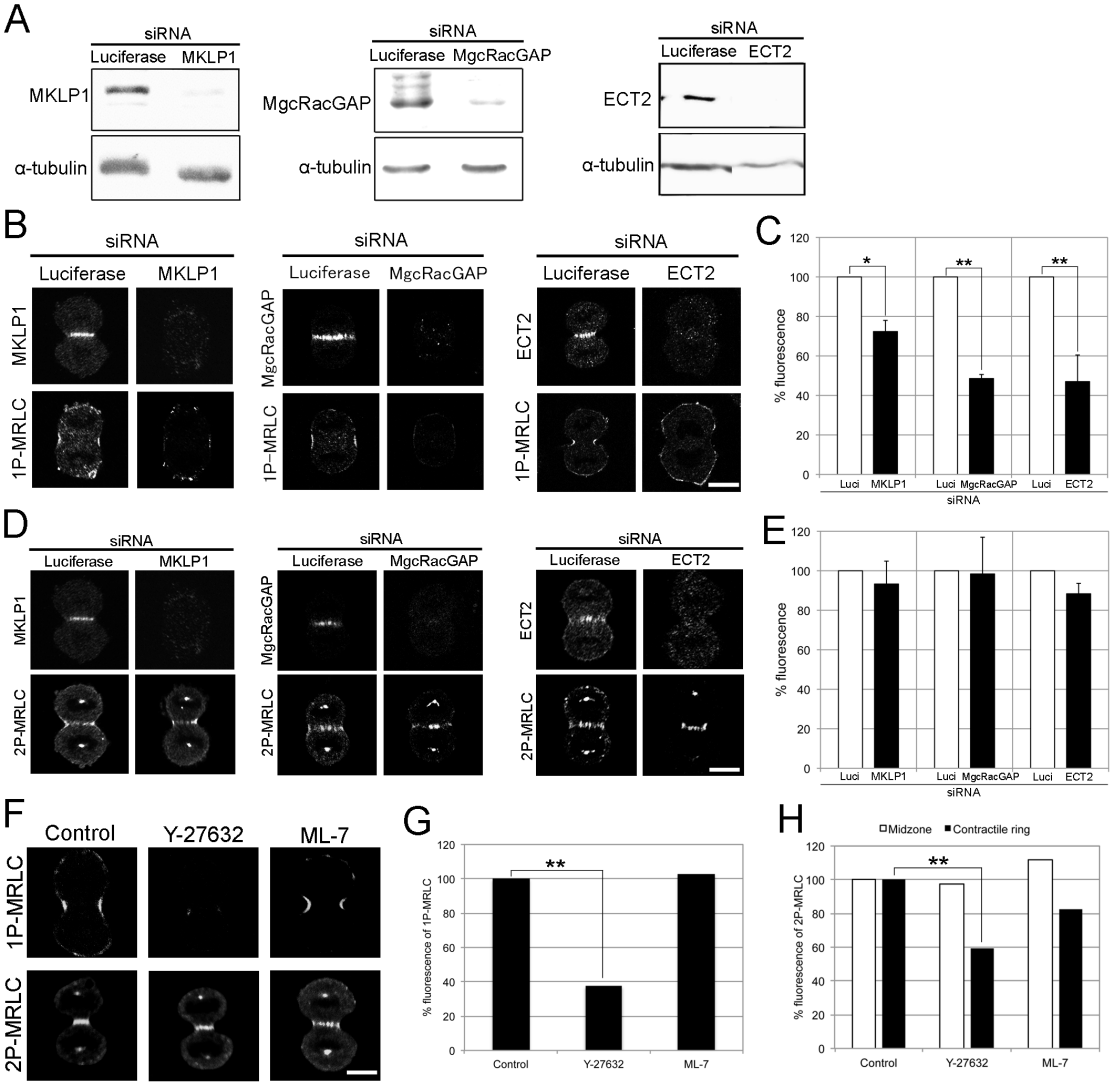
Rho signaling is not required for the localization of 2P-MRLC to the midzone. (A) Western blots for the detection of MKLP1, MgcRacGAP, and ECT2. HeLa cells were transfected with siRNA targeting luciferase, MKLP1, MgcRacGAP, or ECT2, respectively. Loading control: α-tubulin. (B) HeLa cells treated with luciferase, MKLP1, MgcRacGAP, or ECT2 siRNAs were fixed and immunostained with pLC1. Bar, 10 µm. (C) Quantitative analysis of 1P-MRLC fluorescence at the contractile ring in cells depleted of luciferase, MKLP1, MgcRacGAP, or ECT2. The averages of at least 2 independent experiments are shown. Error bars indicate the standard error of the mean (±SEM). *p<0.05, **p<0.01 (*t*-test). *n* ≥15. (D) HeLa cells treated with luciferase, MKLP1, MgcRacGAP, or ECT2 siRNAs were fixed and immunostained as indicated. (E) Quantitative analysis of 2P-MRLC fluorescence at the midzone in cells depleted of luciferase, MKLP1, MgcRacGAP, or ECT2. Error bars indicate ±SEM. (F) HeLa cells treated with 10 µM Y-27632 or 10 µM ML-7 for 30 min were fixed and immunostained with CS1P pAb or 4F12. Bar, 10 µm. (G) Quantitative analysis of 1P-MRLC fluorescence at the contractile ring in cells treated as indicated. Representative data from 2 independent experiments are shown. **p<0.01 (*t*-test). *n* ≥11. (H) Quantitative analysis of 2P-MRLC fluorescence at the midzone (white bar) or contractile ring (black bar) in cells treated as indicated. Representative data from 2 independent experiments are shown. **p<0.01 (*t*-test). *n* ≥17.

Several studies have reported that MKLP1, MgcRacGAP, and ECT2 localize to the midzone in mitotic cells [17]–[19]. Moreover, MKLP1 and MgcRacGAP target ECT2 to the central spindle, leading to Rho GTPase activation at the cell equator [20]–[25] and the subsequent stimulation of Rho-dependent kinases, including Rho kinase and citron kinase, during M-phase. Thus, Rho signaling proteins, including MKLP1, MgcRacGAP, and ECT2, are candidates for the regulator of 2P-MRLC at the midzone. To understand how 2P-MRLC localizes to the midzone, we transfected HeLa cells with 3 kinds of siRNAs targeting MKLP1, MgcRacGAP, and ECT2, respectively. Western blotting showed a clear and specific reduction of the targeted protein in each siRNA-transfected cell line (Fig. 2A). Using immunofluorescence microscopy, we also confirmed the loss of those midzone-localizing proteins and RhoA, at the midzone or the contractile ring in siRNA-transfected cells; this result is in agreement with those of previous studies by other research groups [20]–[22], [24], [25] (Fig. S1). Figure 2B and 2C show that the fluorescence of 1P-MRLC at the contractile ring was significantly altered in cells treated with siRNAs for MKLP1, MgcRacGAP, or ECT2, as reported previously [22], [24]. Interestingly, 2P-MRLC still localized to the midzone in MKLP1-, MgcRacGAP-, or ECT2-depleted cells (Figs. 2D and 2E). To further investigate whether Rho or MLCK signaling controls the localization of 2P-MRLC during cytokinesis, we examined the localization of 2P-MRLC in cells treated with the Rho-kinase inhibitor Y-27632 or the MLCK inhibitor ML-7. As shown in Fig. 2F–H, the presence of 1P-MRLC and 2P-MRLC at the contractile ring was significantly perturbed by Y-27632 treatment, whereas 2P-MRLC was still present at the midzone. Furthermore, the inhibition of MLCK by ML-7 had no significant effect on the localization of 1P- and 2P-MRLC, suggesting that MLCK is not required for the phosphorylation of MRLC during cytokinesis in HeLa cells. These results indicate that the localization of 1P- and 2P-MRLC during cytokinesis in mammalian cells is regulated by at least 2 distinct pathways: (1) Rho signaling mediated by MKLP1, MgcRacGAP, and ECT2 acting through 1P-MRLC and 2P-MRLC at the contractile ring, and (2) a novel Rho-independent pathway involving only 2P-MRLC at the midzone.

### Aurora B is Required for the Localization of 2P-MRLC to the Midzone during Cytokinesis

**Figure 3:**
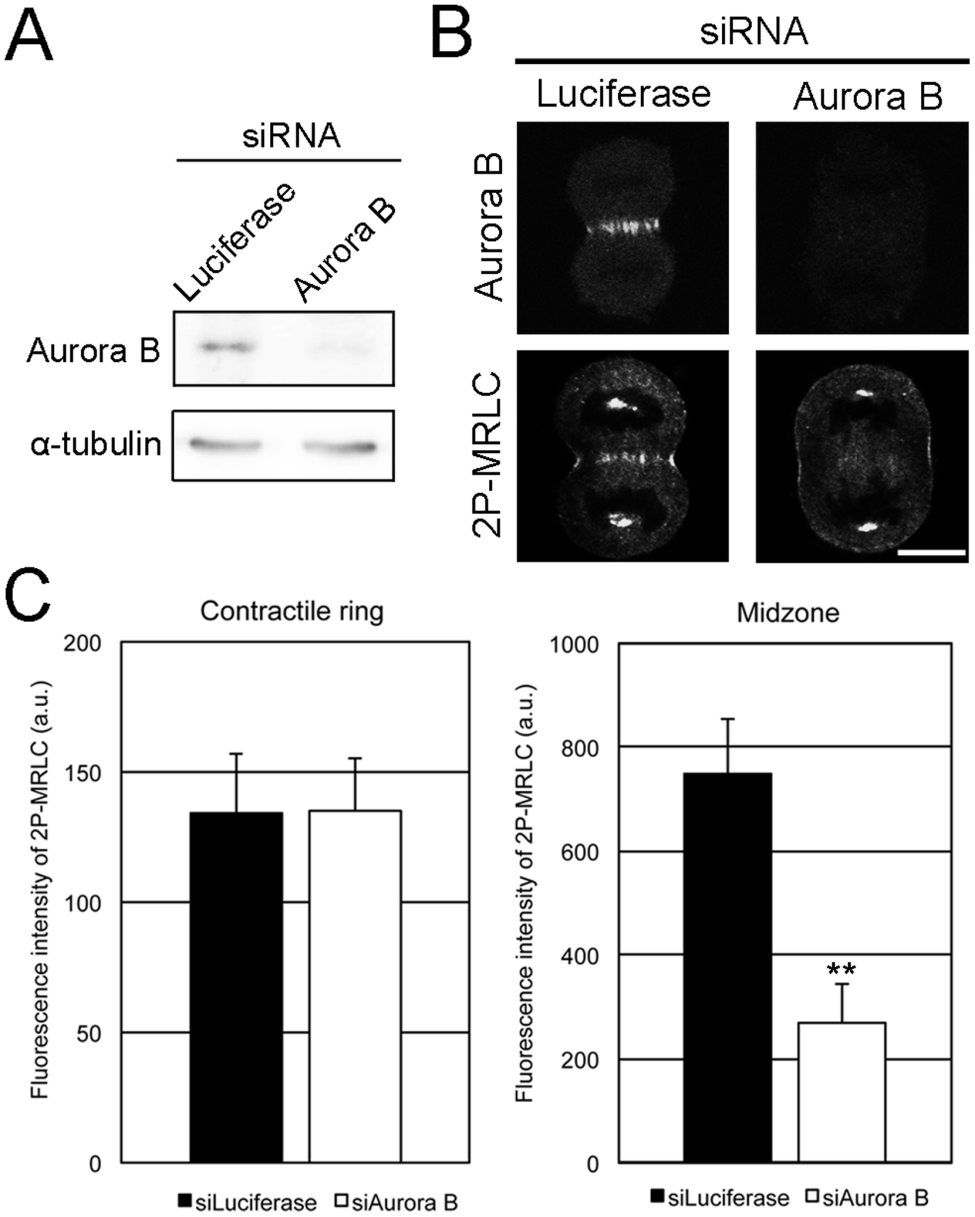
Aurora B is required for the localization of 2P-MRLC to the midzone. (A) Western blots for Aurora B detection. HeLa cells were transfected with siRNAs targeting luciferase or Aurora B. Loading control: α-tubulin. (B) HeLa cells treated with luciferase or Aurora B siRNAs were fixed and immunostained as indicated. Bar, 10 µm. (C) Quantitative analysis of 2P-MRLC fluorescence at the contractile ring (left) or midzone (right) in cells depleted of luciferase or Aurora B. Error bars indicate ±SEM. **p<0.01 (*t*-test). *n* ≥21.

**Figure 4:**
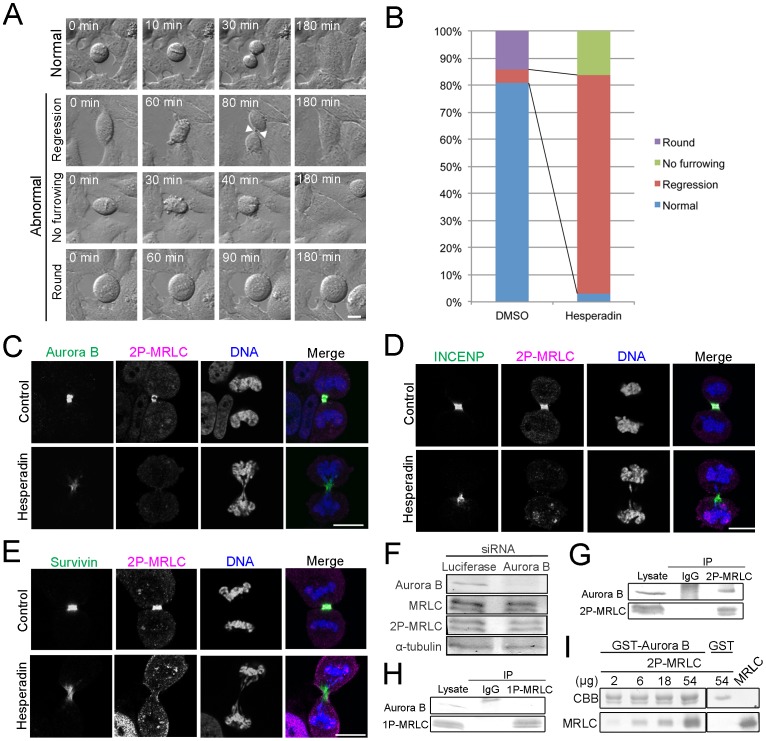
Aurora B activity, but not INCENP or survivin, is crucial for the localization of 2P-MRLC at the midbody. (A) Representative time-lapse imaging of HeLa cells treated with DMSO or 0.1 µM hesperadin for 3 h. Normal: a dividing cell; Regression: a furrow ingression (white arrowheads) but not in cells undergoing abscission; No furrowing: a cell showing no furrow ingression and no abscission; Round: a cell appearing round for at least 3 h. Bar, 10 µm. (B) Quantitative analysis of cells like those shown in (A). *n* ≥63. (C–E) HeLa cells treated with DMSO or 0.1 µM hesperadin for 30 min were fixed and immunostained as indicated. (F) Western blot detection of total MRLC and 2P-MRLC in mitotic HeLa cells treated with siRNA targeting luciferase or Aurora B. (G) Western blot detection of Aurora B in immunoprecipitates of endogenous 2P-MRLC from mitotic HeLa cell lysates. Endogenous 2P-MRLC was immunoprecipitated by 4F12. IP, immunoprecipitates; IgG, normal mouse IgG for a negative control. (H) Western blot detection of Aurora B in immunoprecipitates of endogenous 1P-MRLC from mitotic HeLa cell lysates. Endogenous 1P-MRLC was immunoprecipitated by CS1P mAb. IP, immunoprecipitates; IgG, normal mouse IgG for a negative control. (I) GST-Aurora B and GST were incubated with 2, 6, 18, or 54 µg 2P-MRLC. The upper panel indicates GST-Aurora B (66 kDa) or GST (26 kDa) on a Coomassie Brilliant Blue (CBB)-stained membrane, and the bottom panel shows MRLC detected by western blotting.

Aurora B is involved in various steps of cell division [15], [26]–[28]. Several Aurora B-binding proteins such as inner centromere protein (INCENP) [29], [30] and the anti-apoptosis protein survivin [31]–[33] stimulate Aurora B activity and target the complex to the central spindle. In addition, MRLC is phosphorylated by Aurora B *in vitro* [34], suggesting that Aurora B is a regulator of 2P-MRLC localization at the midzone. To understand how 2P-MRLC localizes to the midzone, cells were transfected with siRNA targeting Aurora B, which significantly reduced Aurora B expression (Fig. 3A). Confocal microscopic observations revealed that the localization of 2P-MRLC to the midzone, but not to the contractile ring, was significantly decreased in Aurora B-depleted cells (Fig. 3B and 3C). Next, we acquired time-lapse images of cells treated with the Aurora B inhibitor hesperadin. We observed 3 types of abnormal cytokinesis: regression, no furrowing, and round (Fig. 4A). Fig. 4B shows that treatment with hesperadin, an Aurora B inhibitor, caused an approximately 17-fold increase in the number of cells showing regression, suggesting that Aurora B activity is required for the completion of abscission. Next, we examined whether Aurora B, INCENP [35], and survivin [36] colocalized with 2P-MRLC at the midbody in cells treated with hesperadin. These 3 proteins, but not 2P-MRLC, still localized to the midbody in hesperadin-treated cells (Fig. 4C-E). These data indicate that Aurora B activity is crucial for the completion of abscission and the localization of 2P-MRLC, but not INCENP and survivin, at the midbody.

To determine how 2P-MRLC is regulated by Aurora B at the midzone and midbody, we investigated whether Aurora B diphosphorylates MRLC in mitotic cells. As shown in Fig. 4F, 2P-MRLC and total MRLC were still detected in Aurora B-depleted mitotic cells, suggesting that Aurora B is not a main kinase for the diphosphorylation of MRLC. Next, we investigated the interaction between 2P-MRLC and Aurora B *in vivo* and *in vitro*. Aurora B co-immunoprecipitated with endogenous 2P-MRLC but not with 1P-MRLC from mitotic HeLa cells extracts (Fig. 4G and 4H), suggesting that Aurora B bound to 2P-MRLC. To examine whether 2P-MRLC directly binds to Aurora B, a pull-down assay was also conducted. As shown in Fig. 4I, 2P-MRLC bound to GST-Aurora B *in vitro*, but not to GST. Taken together, these data suggest that Aurora B is required as a scaffold protein, but not as a kinase, for the localization of 2P-MRLC at the midzone in dividing cells.

### Rho Signaling, but not Aurora B, is Required for the Localization of 1P-MRLC to the Contractile Ring

**Figure 5:**
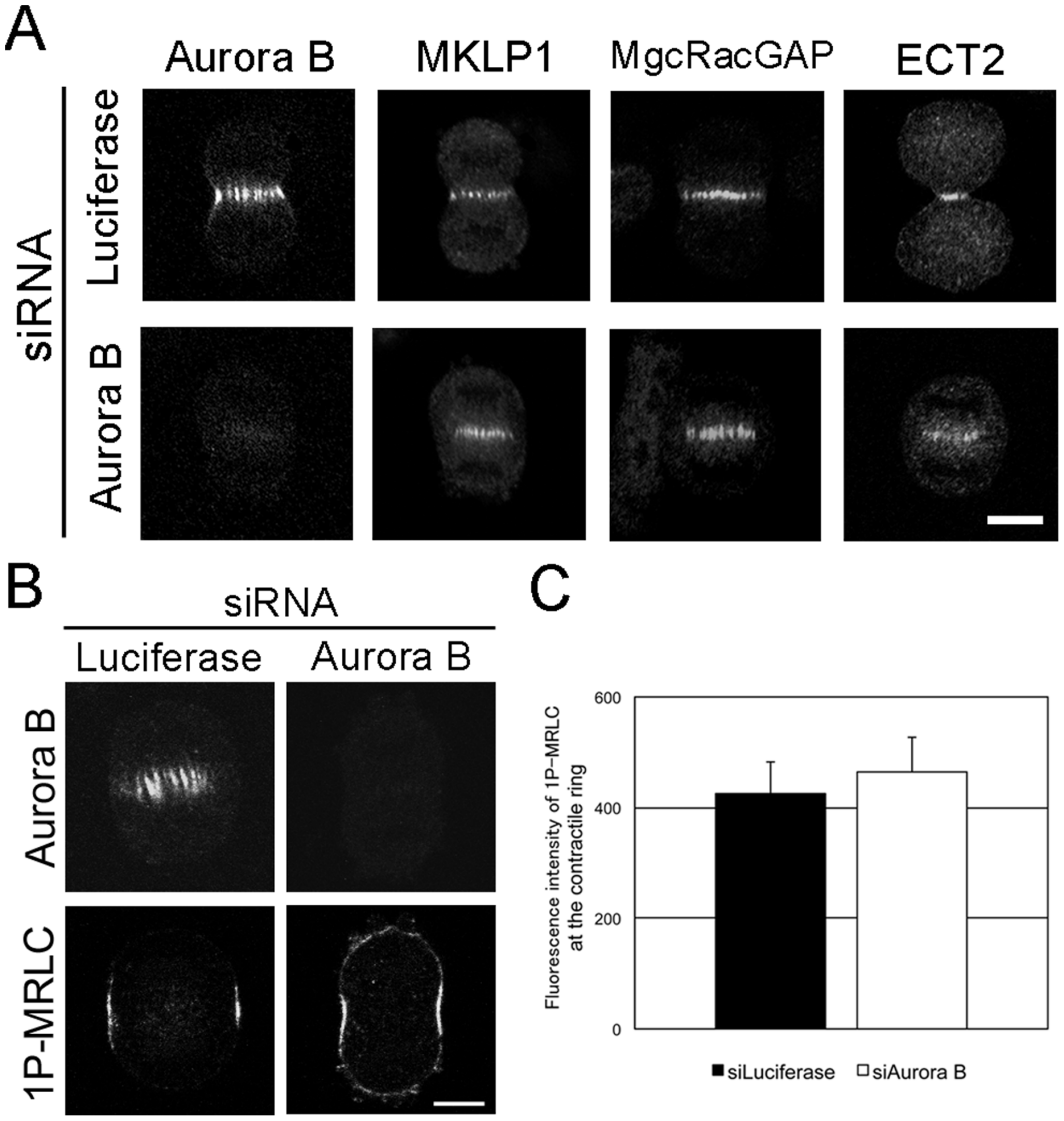
Aurora B is not involved in the localization of 1P-MRLC to the contractile ring. (A and B) HeLa cells treated with either luciferase or Aurora B siRNAs were fixed and immunostained as indicated. Bar, 10 µm. (C) Quantitative analysis of 1P-MRLC fluorescence at the contractile ring in luciferase- or Aurora B-depleted cells. Error bars indicate ±SEM. *n* ≥19.

To determine whether Aurora B is required for the localization of 1P-MRLC at the contractile ring, we transfected HeLa cells with siRNA targeting Aurora B and immunostained cells with MKLP1, MgcRacGAP, and ECT2 antibodies. MKLP1, MgcRacGAP, and ECT2 were still present at the midzone in Aurora B-depleted cells (Fig. 5A). Interestingly, 1P-MRLC also remained at the contractile ring in Aurora B-depleted cells (Figs. 5B and C). These data indicate that 1P-MRLC at the contractile ring is not the downstream target of Aurora B, and this finding is in agreement with the results of previous studies [37], [38].

**Figure 6:**
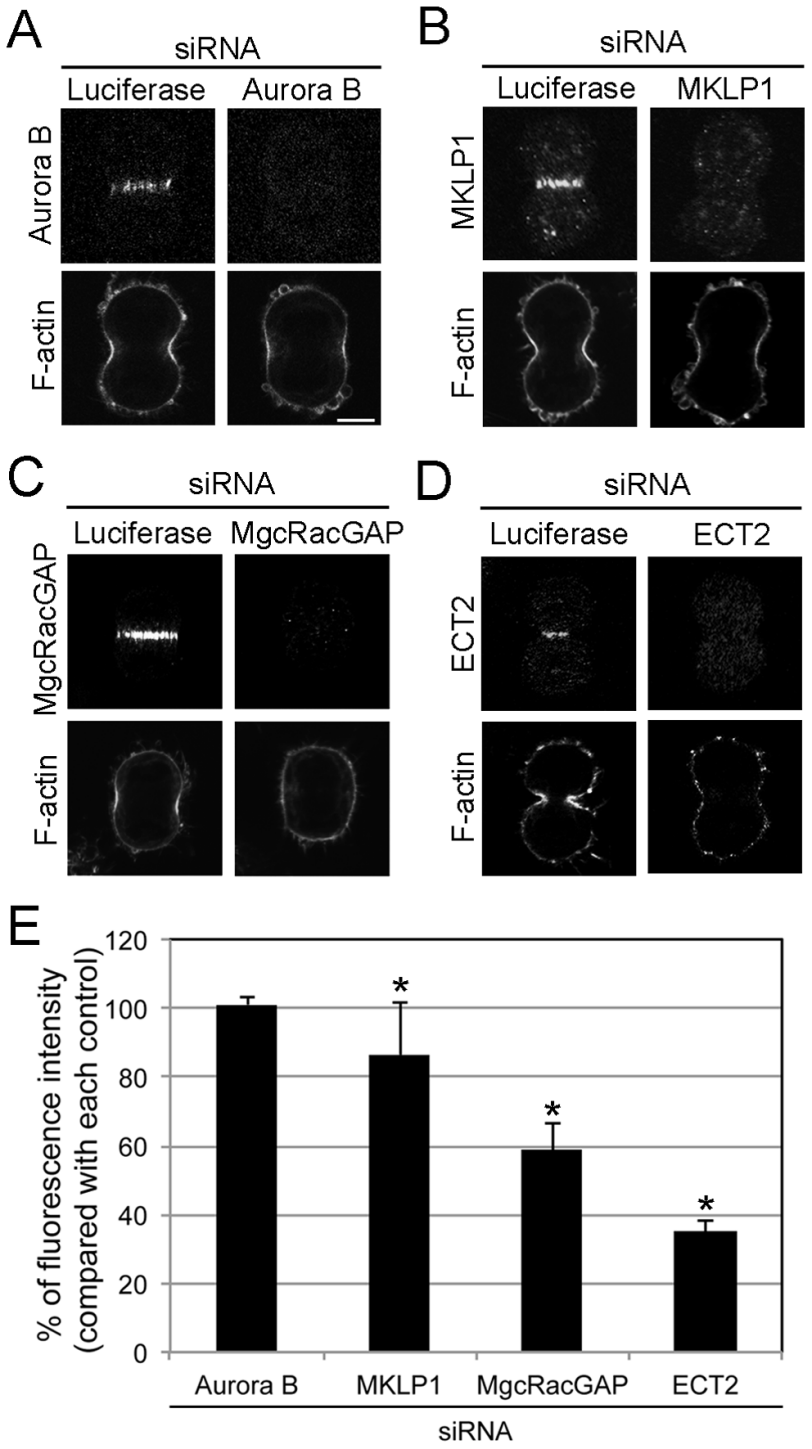
Aurora B and Rho signaling proteins are independent regulators of the localization of F-actin to the contractile ring. HeLa cells treated with siRNAs targeting luciferase, Aurora B (A), MKLP1 (B), MgcRacGAP (C), or ECT2 (D) were fixed and immunostained as indicated. Bar, 10 µm. (E) Quantitative analysis of F-actin fluorescence at the contractile ring in cells treated as indicated. All values are presented as a percentage of the control. Error bars indicate ± SEM. *, p<0.05 (*t*-test). *n* ≥20.

Next, we investigated whether Aurora B and Rho signaling are required for the accumulation of F-actin at the contractile ring. Aurora B-depleted cells showed a normal accumulation of F-actin at the contractile ring (Figs. 6A and E). In contrast, the accumulation of F-actin was inhibited in MKLP1-, MgcRacGAP-, or ECT2-depleted cells (Figs. 6B–E), which is consistent with previous studies [21]. Taken together, these data suggest that Rho signaling, but not Aurora B, is required for the localization of contractile ring components, such as 1P-MRLC and F-actin, during cytokinesis.

## Discussion

**Figure 7:**
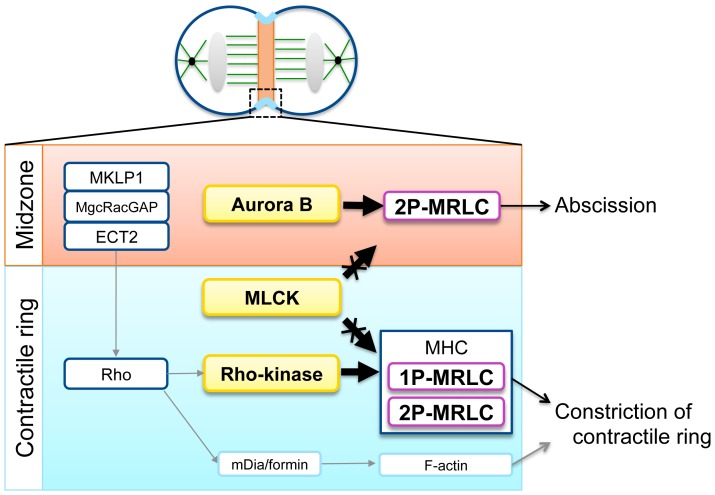
Distinct roles of 1P- and 2P-MRLC during cytokinesis. At the midzone (upper), the localization of 2P-MRLC is controlled by Aurora B and is essential for cell abscission. Moreover, at the contractile ring (lower), Rho signaling proteins, including MKLP1, MgcRacGAP, ECT2, Rho, and Rho kinase, but not MLCK and Aurora B, regulate the localization of 1P-MRLC, 2P-MRLC, and F-actin during cytokinesis, and they are essential for constriction of the contractile ring. The localization of MLCK and Rho kinase was described according to the literature ([40] and [68], respectively). The Rho-dependent regulation of F-actin by mDia/formin was performed as previously described [69].

Our previous study reported that 2P-MRLC, but not 1P-MRLC, localizes at the contractile ring and at the midzone during cytokinesis [14]. Because MHC and F-actin were not observed at the midzone, this finding first suggested that 2P-MRLC does not function as a subunit of myosin II at the midzone. It has been shown that Rho signaling is required for the localization of 1P-MRLC at the contractile ring [22], [24]. However, the regulation of the localization of 2P-MRLC during cytokinesis remains to be clarified. Here, we determined the signaling pathways required for the localization of 2P-MRLC in dividing HeLa cells. Our data revealed that the localization of 2P-MRLC at the contractile ring, but not at the midzone, was regulated by the Rho signaling pathway, whereas its localization at the midzone was regulated by the Aurora B pathway (Fig. 7).

Many kinases localized at the constricted area in dividing cells have been identified as MRLC kinases, such as MLCK [39], [40], Rho kinase [41]–[43], citron kinase [44], and Aurora B [34]. Biochemical analyses also revealed that, except for Aurora B, these kinases can mono- and diphosphorylate MRLC *in vitro* [9], [10], [41], [43], [44]. The present data showed that MLCK signaling is not required for the localization of 1P- and 2P-MRLC to the constricted area of dividing HeLa cells. A previous microinjection experiment showed that the activity of MLCK affects mitosis but not cytokinesis [45]. A study using microarray technology revealed that *MLCK* gene expression was low in various tumors, compared with its expression in non-tumors [46]. Recently, Wu *et al.* confirmed this by western blotting using MLCK antibodies [47]. These results suggest that the contribution of MLCK to MRLC phosphorylation might be small in cytokinesis, particularly in carcinoma cells, such as HeLa cells. Previously, we showed that the accumulation of citron kinase and 1P-MRLC at the equatorial region was inhibited in ECT2-, MgcRacGAP-, or MKLP1-depleted cells [24]. Treatment with C3 exoenzyme, a RhoA inhibitor, also inhibited the accumulation of RhoA and 1P-MRLC at the equatorial region of mitotic cells [24]. These results indicate that citron kinase and the Rho signaling pathway are required for the localization of 1P-MRLC in the furrowing region. However, we did not address the localization of 2P-MRLC in those experiments. Further, because a conventional microscope was used to observe the localization of these proteins at the equatorial region of mitotic cells, it was not clear whether RhoA and/or Rho-dependent kinases localized at the contractile ring and/or the midzone in dividing cells. Thus, further study using confocal microscopy was required to elucidate how these proteins interact with phosphorylated MRLC at the constricted area in dividing cells.

Here, we demonstrated that Aurora B binds directly to 2P-MRLC. MRLC has been shown to associate with MHC via 2 IQ motifs in the neck region linking the head and tail domains in myosin II [48], [49]. Analysis with Clustal Omega (EMBL-EBI) indicated that Aurora B and another MRLC-binding protein, MIR [50], have no IQ motifs in their amino acid sequences (data not shown). In these proteins, MRLC-binding site(s) other than the IQ motif need to be elucidated.

The kinase required for the diphosphorylation of MRLC in mitotic cells is still unknown. We have previously reported that zipper-interacting protein (ZIP) kinase (also known as DAPK3/Dlk) diphosphorylates MRLC *in vitro* and *in vivo* [51], [52]. Although another group has reported that GFP-tagged ZIP kinase localizes at the contractile ring [53], the localization of endogenous ZIP kinase has not been established. Further work is required to elucidate the role of ZIP kinase in the diphosphorylation of MRLC at the midzone.

Aurora B activity is required for the phosphorylation of MKLP1 [54], [55] and MgcRacGAP [56] at the midbody and for protection against tetraploidization [57], suggesting that Aurora B plays an important role in correct abscission. Although various models of abscission in animal cells have been proposed [58]–[60], the exact mechanism remains controversial. In this manuscript, 2P-MRLC was newly identified as a midzone-localizing, Aurora B-binding protein. What is the role of the 2P-MRLC/Aurora B complex during cytokinesis? Two midzone-localizing and Aurora B-binding proteins, INCENP [35] and MKLP2 [61], have been identified as a regulator of Aurora B activity and a translocator of Aurora B, respectively. To clarify the role of 2P-MRLC as an Aurora B-binding protein, we compared the amino acid sequence of MRLC with INCENP and MKLP2 using Clustal Omega. However, there were no domains highly conserved between MRLC and these 2 proteins (data not shown), suggesting that 2P-MRLC has a novel activity as an effector of Aurora B at the midzone. Whether the 2P-MRLC/Aurora B complex regulates the function of other midzone-localizing, Aurora B-binding proteins at the midzone during abscission remains to be elucidated.

## Materials and Methods

### Reagents

The following antibodies and peptides were purchased from commercial sources: mouse anti-MRLC monoclonal antibody (mAb) (E-4), rabbit anti-MKLP1 polyclonal antibody (pAb), rabbit anti-ECT2 pAb, mouse anti-RhoA mAb, rabbit anti-survivin pAb, and normal mouse IgG from Santa Cruz Biotechnology, Inc. (Santa Cruz, CA, USA); mouse anti-Aurora B mAb from BD Biosciences (San Jose, CA, USA); rabbit anti-Aurora B pAb from Abcam (Cambridge, UK); FITC- or TRITC-phalloidin from Sigma Aldrich (St. Louis, MO); mouse anti-α-tubulin mAb (DM1A) from Cedarlane Labs (Hornby, Ontario, Canada); rabbit anti-pSer19 MRLC (CS1P) pAb, mouse anti-pSer19 MRLC (CS1P) mAb, mouse anti-pSer19 and pThr18 MRLC (CS2P) pAb, and rabbit anti-INCENP pAb from Cell Signaling Technology (Beverly, MA, USA); alkaline phosphatase conjugated anti-mouse and anti-rabbit secondary antibodies from Promega KK (Tokyo, Japan); and Alexa Fluor 488 or 568-conjugated anti-mouse or anti-rabbit IgG (H+L) from Molecular Probes. Rabbit anti-MgcRacGAP pAb [24] and mouse anti-2P-MRLC mAb (4F12) [14] have been previously described. The following chemicals were purchased from commercial sources: calyculin A from Wako Pure Chemical Industries (Osaka, Japan); pepstatin A from Peptide Institute (Osaka, Japan); nocodazole, thymidine, ML-7, and poly-l-lysine from Sigma Aldrich (St. Louis, MO, USA); and 4′,6-diamidino-2-phenylindole from Nacalai Tesque (Kyoto, Japan). Y-27632 and hesperadin [62] were provided by Welfide Corporation (Osaka, Japan) and Boehringer Ingelheim Austria GmbH, respectively.

### Cell Culture and Cell Synchronization

HeLa (human cervical carcinoma) cells were cultured as previously described [63]. Mitotic HeLa cells were obtained by the double thymidine/nocodazole arrest procedure performed under sterile conditions as follows. Cells were grown with 2 mM thymidine for 24 h, without thymidine for another 12 h, with 2 mM thymidine again for 12 h, and finally without thymidine for 5 h. Cells were then exposed to 40 ng/mL nocodazole for 3 h. Rounded cells were collected by mechanical shake-off and pipetting, incubated in a poly-l-lysine-coated polystyrene dish (BD Falcon, Franklin Lakes, NJ) for 1 h in 40 ng/mL nocodazole, and then cultured in fresh medium for another 1 h.

### RNAi

siRNAs targeting luciferase, MKLP1, MgcRacGAP, ECT2, or Aurora B (NIPPON EGT, Toyama, Japan) were used to knockdown the expression of their respective cognate proteins. The targeting sequences of luciferase (5′-CGUACGCGGAAUACUUCGAdTdT-3′), MKLP1 (5′-GCAGUCUUCUAGGUCAUCUdTdT-3′), MgcRacGAP (5′-CCUCUUCUGACCUUUCGCCdTdT-3′), ECT2 (5′-GCUUGGGAAAGGCGGAAUGdTdT-3′), and Aurora B (5′-GGUGAUGGAGAAUAGCAGUdTdT-3′) were designed as previously described [24], [61], [62]. HeLa cells were transfected with 300 pmol of each siRNA using Oligofectamine (Invitrogen, Carlsbad, CA, USA) and then incubated for 24–48 h. The knockdown efficiency was determined by western blotting as previously described [64].

### Immunostaining, Microscopy, and Image Analysis

Immunostaining was performed as previously described [63] except for the staining of 2P-MRLC and RhoA. 2P-MRLC was stained with 4F12 as previously described [14]. In the absorption experiments illustrated in Fig. 1, 33 ng/mL 4F12 in 1% BSA/PBS was preincubated with 20 µg/mL 0P-MRLC, 20 µg/mL 2P-MRLC, or PBS at 37°C for 60 min before the treatment to fixed cells. RhoA was stained as previously described [65]. The specimens were observed with either an LSM410 microscope (Carl Zeiss) with a Plan-NEOFLUAR 100× NA 1.3 oil immersion objective or a FLUOVIEW FV1000-D microscope with a UPLSAPO 60× NA 1.4 or UPLSAPO 100× NA 1.4 oil immersion objective (Olympus, Tokyo, Japan). Immunofluorescence intensity was measured from the obtained images (640×640 pixels) by using ImageJ (NIH, Bethesda, MD, USA) (Table S1). The dynamic range of intensity in the fluorescence image was set from 0 to 255 in arbitrary units. The thickness of the contractile ring was defined from the width of the cell cortex stained by phalloidin (approximately 0.6 µm). The width of the midzone was defined from the width of the cell diameter subtracted by the width of the contractile ring. For time-lapse imaging, HeLa cells were cultured in a glass-based dish (Asahi Glass, Tokyo, Japan). After 1 day, cells were observed by using FV1000-D microscope as previously described [66]. Differential interference contrast microscopic images were acquired every minute for 3 h. All acquired images were processed with Adobe Photoshop.

### Protein Expression, Purification, and *in vitro* Phosphorylation of MRLC

The expression and purification of GST-MRLC2 and GST-ZIPK were carried out as previously described [14]. GST-Aurora B was expressed in *Escherichia coli* BL21 in 2× YT medium (10 mg/mL Bacto yeast extract, 16 mg/mL Bacto tryptone, 5 mg/mL NaCl) containing 100 µg/mL ampicillin at 37°C with vigorous agitation. Protein expression was induced by adding IPTG to 0.4 mM, and the cells were incubated for 12 h at 25°C. Protein bound to Glutathione-Sepharose 4B (GE Healthcare) was suspended in HNTM buffer (50 mM HEPES-NaOH, pH 7.2; 150 mM NaCl; 0.1% [vol/vol] Triton X-100; 1 mM MgCl2; 1 mM DTT; 1 mM PMSF; 1 µg/mL pepstatin A) as previously described [61]. To remove the GST-tag, GST-MRLC was incubated with PreScission Protease (GE Healthcare) according to the manufacturer’s procedure. The protein concentration was determined by Lowry assay (Sigma-Aldrich, St. Louis, MO, USA) or Bradford assay (Bio-Rad Laboratories, Hercules, CA, USA). Phosphorylation of MRLC by GST-ZIPK was carried out at 37°C for 2 h in reaction buffer I (2.3 mM Tris-HCl, pH 7.8; 113 mM NaCl; 2 mM MgCl_2_; 1.2 mM KH_2_PO_4_; 6.6 mM Na_2_PO_4_) with 0.1 mg/mL MRLC2, 1 mM ATP or water, and 10 µg/ml GST-ZIPK. Phosphorylation of MRLC by MLCK was carried out at 25°C for 2 h in reaction buffer II (21 mM HEPES-NaOH, pH 7.2; 7 mM Tris-HCl, pH 7.2; 94 mM NaCl; 1.5 mM MgCl_2_; 2.0 mM CaCl_2_; 0.3 mM EGTA; 0.6 mM DTT; 0.5 mM PMSF; 0.4 µg/mL pepstatin A) with 50 µg/mL calmodulin (Sigma Chemical, St. Louis, MO, USA), 2.0 mg/mL MRLC, 1 mM ATP or water, and 100 µg/mL MLCK. Diphosphorylation of MRLC in the sample was confirmed by urea/glycerol-PAGE [67].

### *In vivo* Phosphorylation of MRLC, Immunoprecipitation, and *in vitro* Binding Assay

*In vivo* phosphorylation of MRLC was determined as follows. After incubation with siRNA targeting Aurora B or luciferase for 48 h, cells were collected by pipetting in PBS containing 0.1 µM calyculin A. Cells were then centrifuged at 500×*g* for 5 min at 4°C, and the cell pellets were homogenized with Laemmli sample buffer. The extracts were subjected to sodium dodecyl sulfate polyacrylamide-gel electrophoresis and western blotting. Immunoprecipitation and the *in vitro* binding assay were carried out as follows. The mitotic cells, collected as described above, were lysed by sonication in IP buffer (20 mM Tris-HCl, pH 8.0; 150 mM NaCl; 0.5% NP-40; 5 mM EGTA; 10 mM MgCl_2_; 0.1 µM calyculin A; 1 mM PMSF; 5 µg/mL pepstatin A). The extract was centrifuged at 15,100×*g* for 30 min at 4°C, and the supernatant was collected. Immunoprecipitation was carried out using normal mouse IgG, 4F12, or CS1P mAb. After the mixtures were agitated for 1.5 h at 4°C, Protein G-Sepharose (GE Healthcare) was added. The mixtures were incubated for another 1.5 h at 4°C, centrifuged at 700×*g* for 2 min at 4°C, and washed 3 times with IP buffer. The beads were collected and subjected to SDS-PAGE. Western blotting was performed as previously described [64]. GST-Aurora B bound to Glutathione-Sepharose (5 µL) was incubated with 2–54 µg 2P-MRLC for 1 h at 4°C in binding buffer (45 mM HEPES-NaOH, pH 7.2; 1 mM Tris-HCl, pH 7.2; 139 mM NaCl; 1 mM MgCl_2_; 0.3 mM CaCl_2_; 1 mM DTT; 1 mM PMSF; 2.6 µg/mL pepstatin A; 0.1% Triton X-100; 6.8 µg/mL CaM) with 14 µg/mL MLCK. The beads were washed 3 times with excess HNTM buffer, and the bound MRLC was analyzed by western blotting.

## Supporting Information

Figure S1 **Localization of Rho-signaling proteins in siRNA-treated cells.** Cells treated with siRNAs specific for either luciferase, MKLP1, MgcRacGAP, or ECT2 were fixed and reacted with the indicated antibodies against the Rho-signaling proteins (MKLP1, MgcRacGAP, ECT2, and RhoA). Bar, 10 µm.
(TIF)

Table S1 **Quantitative analysis of the fluorescence intensity of F-actin, phosphorylated MRLC, MHC, and Rho-signaling proteins in cells depleted of Rho-signaling proteins or Aurora B.** Each value represents the relative fluorescence intensity of the indicated proteins in cells treated with MKLP1, MgcRacGAP, ECT2, or Aurora B siRNAs. Numbers in red indicate a significant difference compared with each control (p<0.05, *t*-test).
(DOC)
