# Supplementary figures and images for: Aurora B but Not Rho/MLCK Signaling Is Required for Localization of Diphosphorylated Myosin II Regulatory Light Chain to the Midzone in Cytokinesis
Source: PLoS One. 2013 Aug 7;8(8):e70965. doi: 10.1371/journal.pone.0070965 (PMC3737224; doi:10.1371/journal.pone.0070965)

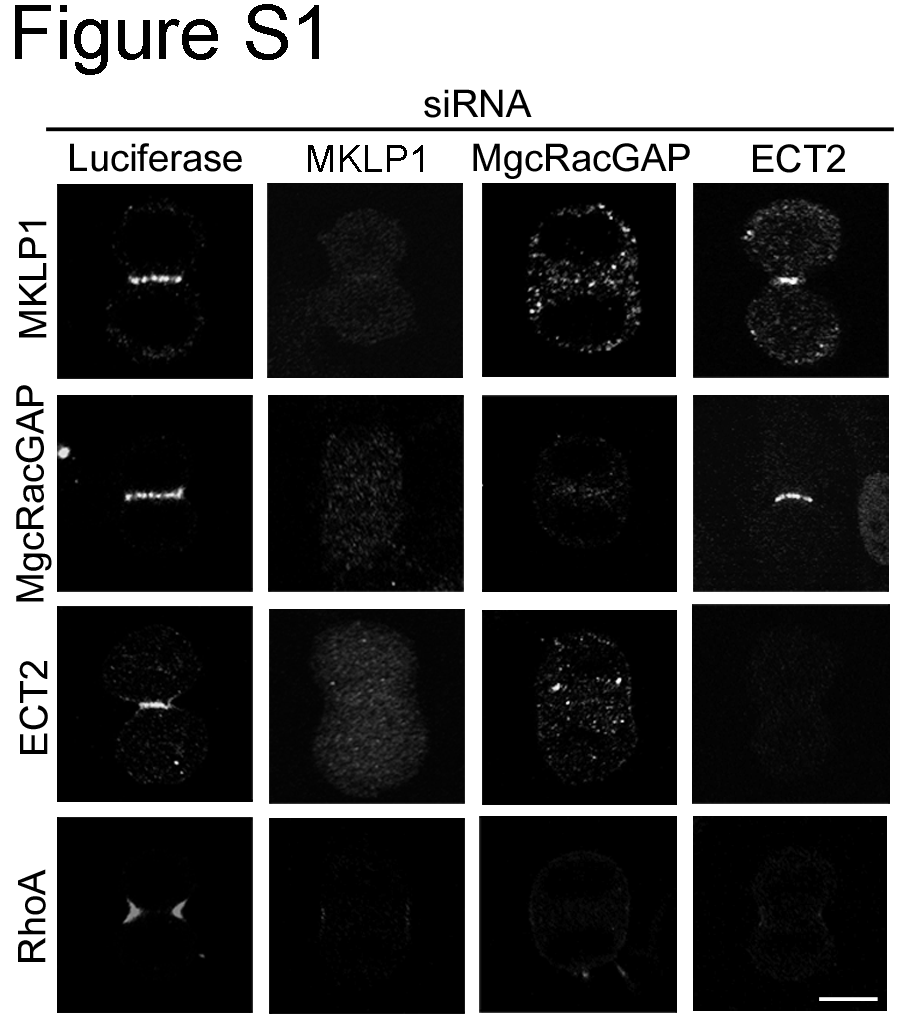

Supplement: Figure S1 — Localization of Rho-signaling proteins in siRNA-treated cells. Cells treated with siRNAs specific for either luciferase, MKLP1, MgcRacGAP, or ECT2 were fixed and reacted with the indicated antibodies against the Rho-signaling proteins (MKLP1, MgcRacGAP, ECT2, and RhoA). Bar, 10 µm. (TIF) [file pone.0070965.s001.tif]
