# Supplementary material for: Aurora B but Not Rho/MLCK Signaling Is Required for Localization of Diphosphorylated Myosin II Regulatory Light Chain to the Midzone in Cytokinesis
Source: PLoS One. 2013 Aug 7;8(8):e70965. doi: 10.1371/journal.pone.0070965 (PMC3737224; doi:10.1371/journal.pone.0070965)
Supplement: Table S1 — Quantitative analysis of the fluorescence intensity of F-actin, phosphorylated MRLC, MHC, and Rho-signaling proteins in cells depleted of Rho-signaling proteins or Aurora B. Each value represents the relative fluorescence intensity of the indicated proteins in cells treated with MKLP1, MgcRacGAP, ECT2, or Aurora B siRNAs. Numbers in red indicate a significant difference compared with each control (p<0.05, t-test). (DOC) [file pone.0070965.s002.doc]

Table S1. Quantitative analysis of fluorescent intensity

|  | RNAi | | | | | | | | |
| --- | --- | --- | --- | --- | --- | --- | --- | --- | --- |
|  | **MKLP1** | | **MgcRacGAP** | | **ECT2** | | **Aurora B** | | |
|  | % | n | % | n | % | n | % | | n |
| **MKLP1** | Midzone |  | Midzone |  | Midzone |  | midzone | |  |
| 1 | 42.6 | 24 | 10.1 | ≥22 | 101 | ≥18 | 112 | | ≥20 |
| 2 | 52.4 | 25 |  |  |  |  |  | |  |
| 3 | 36.8 | 25 |  |  |  |  |  | |  |
| **Average** | **43.9** |  | **10.1** |  | 101.0 |  | 112.0 | |  |
| **MgcRacGAP** | Midzone |  | Midzone |  | Midzone |  | midzone | |  |
| 1 | 30.3 | ≥20 | 9.4 | ≥22 | 70.5 | ≥14 | 106 | | ≥22 |
| 2 |  |  | 15.8 | ≥25 | 85.8 | ≥15 |  | |  |
| 3 |  |  |  |  |  |  |  | |  |
| **Average** | **30.3** |  | **12.6** |  | 78.2 |  | 106.0 | |  |
| **ECT2** | Midzone |  | Midzone |  | Midzone |  | midzone | |  |
| 1 | 36.9 | ≥20 | 34.8 | 20 | 5.31 | ≥20 | 112 | | ≥22 |
| 2 |  |  |  |  | 1.28 | ≥18 |  | |  |
| 3 |  |  |  |  | 16.91 | ≥29 |  | |  |
| 4 |  |  |  |  | 8.23 | ≥30 |  | |  |
| **Average** | **36.9** |  | **34.8** |  | **7.9** |  | 112.0 | |  |
| **RhoA** | Contractile ring |  | Contractile ring |  | Contractile ring |  | Contractile ring | |  |
| 1 | 46.7 | ≥20 | 57.7 | ≥25 | 45.4 | ≥12 | No data | |  |
| 2 |  |  |  |  | 51 | ≥9 |  | |  |
| 3 |  |  |  |  | 40.5 | ≥17 |  | |  |
| **Average** | **46.7** |  | **57.7** |  | **45.6** |  |  | |  |
| **F-actin** | Contractile ring |  | Contractile ring |  | Contractile ring |  | Contractile ring | |  |
| 1 | 101.4 | 25 | 51.2 | ≥24 | 27.4 | ≥20 | 103 | | 20 |
| 2 | 71.5 | 21 | 66.2 | ≥21 | 36.22 | ≥22 | 104 | | ≥28 |
| 3 |  |  |  |  | 34.71 | ≥29 | 96 | | ≥26 |
| 4 |  |  |  |  | 41.5 | ≥30 |  | |  |
| **Average** | **71.5** |  | **66.2** |  | **37.5** |  | 101.0 | |  |
| **1P-MRLC** | Contractile ring |  | Contractile ring |  | Contractile ring |  | Contractile ring | |  |
| 1 | 78 | 25 | 46.5 | ≥23 | 96.8 | 15 | 109 | | ≥19 |
| 2 | 66.7 | 21 | 50.6 | ≥21 | 53.5 | ≥20 | 105 | | ≥25 |
| 3 |  |  |  |  | 29.2 | 20 | 102 | | ≥23 |
| 4 |  |  |  |  | 32.8 | ≥25 |  | |  |
| 5 |  |  |  |  | 22.7 | ≥21 |  | |  |
| **Average** | **72.35** |  | **48.55** |  | **47** |  | 105.3 | |  |
| **2P-MRLC** | Midzone |  | Midzone |  | Midzone |  | Midzone | Contractile ring |  |
| 1 | 81.7 | 25 | 79.8 | ≥35 | 96.7 | 20 | 36.1 | 100.3 | ≥21 |
| 2 | 104.9 | 21 | 117 | ≥20 | 80 | 20 | 42.7 | 90.3 | ≥28 |
| 3 |  |  |  |  |  |  | 30.2 | 109.4 | ≥26 |
| **Average** | 93.3 |  | 98.4 |  | 88.35 |  | **36.3** | 100 |  |
| **pan-MHC** | Contractile ring |  | Contractile ring |  | Contractile ring |  | Contractile ring | |  |
| 1 | No data |  | No data |  | 73.7 | ≥23 | No data | |  |
| 2 |  |  |  |  | 96.74 | ≥24 |  |  |  |
| 3 |  |  |  |  | 72.91 | ≥17 |  |  |  |
| **Average** |  |  |  |  | 81.1 |  |  |  |  |
| **MHC IIA** | Contractile ring |  | Contractile ring |  | Contractile ring |  | Contractile ring | |  |
| 1 | No data |  | No data |  | 7.6 | ≥21 | No data | |  |
| 2 |  |  |  |  | 41.45 | ≥22 |  |  |  |
| 3 |  |  |  |  | 45.3 | ≥20 |  |  |  |
| **Average** |  |  |  |  | **31.5** |  |  |  |  |
| **MHC IIB** | Contractile ring |  | Contractile ring |  | Contractile ring |  | Contractile ring | |  |
| 1 | No data |  | No data |  | 24.9 | ≥17 | No data | |  |
| 2 |  |  |  |  | 42.77 | 20 |  |  |  |
| 3 |  |  |  |  | 31.42 | ≥25 |  |  |  |
| **Average** |  |  |  |  | **33.0** |  |  |  |  |
| **Aurora B** | Midzone |  | Midzone |  | Midzone |  | midzone | |  |
| 1 | No data |  | 95.9 | 20 | No data |  | No data | |  |
| 2 |  |  |  |  |  |  |  |  |  |
| 3 |  |  |  |  |  |  |  |  |  |
| **Average** |  |  | 95.9 |  |  |  |  |  |  |
